# Anterior migration of Ozurdex implant: a review on risk factors, complications, and management

**DOI:** 10.1186/s40942-023-00513-5

**Published:** 2023-11-27

**Authors:** Panagiotis Tsoutsanis, Dimitrios Kapantais

**Affiliations:** Northern Care Alliance NHS Foundation Trust, Rochdale Eye Unit, Rochdale Infirmary, Greater Manchester, UK

**Keywords:** Ozurdex, Intravitreal implant, Macular edema, Anterior migration of ozurdex

## Abstract

**Purpose:**

To describe the common risk factors, complications, and management options for anterior migration of Ozurdex implant.

**Methods:**

A comprehensive review of the literature was performed.

**Results:**

Amongst the most common risk factors predisposing to implant anterior migration we found a history of pseudophakia or aphakia or previous vitrectomy. The most common complication is that of corneal edema.

**Conclusions:**

A variety of management options to treat migration of the dexamethasone implant are utilized by different specialists around the world. These depend on the doctor’s preference, presence of corneal damage and history of previous migrations after repositioning the implant. The most common approaches are operative or non-operative implant repositioning and surgical implant removal.

## Introduction

Ozurdex^®^ is a 0.46 mm in diameter and 6 mm in length biodegradable dexamethasone implant that uses the NOVADUR^®^ drug delivery technology, in which a biodegradable material is paired with an active drug. The implant is injected within the vitreous where it slowly releases 0.7 mg of dexamethasone. It is being used to treat macular edema secondary to pathologies such as branch or central retinal vein occlusion (BRVO/CRVO), diabetic macular edema and non-infectious posterior uveitis [1–3].

An uncommon but significant complication of Ozurdex^®^ use is its migration to the anterior chamber of the eye. The risk factors of this complication include previous complicated intraocular lens (IOL) implantation, previous vitrectomy, iris reconstruction, aphakic or pseudophakic eyes, zonular dehiscence and open or defective lens capsule [3–9]. If the implant is not repositioned or removed in due course, this anterior migration can lead to increased intraocular pressure and corneal edema. In case corneal edema does not resolve the only management option to restore vision can often be a keratoplasty [2, 10, 11].

## Methods

A comprehensive literature search was conducted in November 2023 using PubMed, Google Scholar, ScienceDirect and was limited to studies published in English and until November 2023. The search strategy used the following terms: ozurdex migration, dexamethasone implant migration, anterior displacement of ozurdex. We found 38 relevant papers (case-reports, research articles and case-series) published in peered reviewed journals.

## Results

Out of the 59 patients reviewed, 60% were male, and 40% were female. The average age of the patients was 66 years, indicating that this issue can affect individuals across different age groups.

Most patients (98%) had at least one established risk factor for implant migration in the eye, with a history of pseudophakia and/or vitrectomy being the most common. Other factors included posterior vitrectomy, iris claw lens, iris sutured IOL, and various complications associated with prior ocular procedures.

A significant majority (83%) of the patients experienced serious complications following anterior migration of the Ozurdex implant. These complications primarily included corneal edema, which can severely impact visual acuity and patient comfort. The remaining 17% showed either no complications or only a mild rise in intraocular pressure (IOP).

Various management options were employed to address the complications resulting from anterior migration. The most common approach was surgical removal, chosen in 54% of the cases. Surgical repositioning, whether through surgical techniques (27%) or non-surgical means (14%) like postural maneuvering and ocular massage, was utilized in 41% of the cases. Only 5% of the patients followed a “watch and wait” approach, with the implant eventually dissolving on its own.

Table 1 below shows the findings of the literature review regarding the ophthalmic history of the affected eyes, complications after anterior migration and final management.

**Table 1:** Literature review

Citation	Demographics	Major ophthalmic history at the time of Ozurdex placement (intravitreal injections not included)	AC complications due to anterior migration	Management post-migration
Stepanov et al. [1]	Male, 65 years old	PPV in pseudophakic patient with iris claw lens, Basal iridectomy	High IOP Corneal edema	Implant removal via anterior chamber washout, no need for corneal transplantation
Pardo-Lopez et al. [2]	Male, 68 years old	Posterior capture rupture, vitrectomy, BRVO, Iris-claw IOL	Corneal edema	Surgical removal and corneal transplantation
Zafar et al. [3]	Male, 74 years old	Anterior vitrectomy, IOL iris sutured and Descemet stripping automated endothelial keratoplasty	No complications	Watch & wait. Implant dissolved
Glidai et al. [4]	Male, 56 years old	BRVO, congenital inferior iris coloboma not involving the choroid, posterior chamber IOL, previous uncomplicated ozurdex implantation	Corneal edema	Surgical removal. Balanced salt solution (BSS) were used to guide the implant out of the anterior chamber
Gullapalli et al. [10]	Patient 1: Female, 73 years old Patient 2: Female, 69 years old	Patient 1: lensectomy, posterior capsular tear, aphakia, vitrectomy Patient 2: posterior capsular tear, AC IOL, Ahmed tube shunt, PPV	Patient 1: Corneal edema Patient 2: corneal edema	Patient 1: surgical removal. Lens glide was used with injection of BSS Patient 2: surgical removal and keratoplasty
Jamshidi et al. [12]	Male, 87 years old	Multiple IOLs replaced, multiple previous ozurdex implants, intrascleral haptic fixation IOL, DSAEK and vitrectomy	No complications	Surgical removal as a precaution
Jonas et al. [13]	Male, 52 years old	Vitrectomy, Aphakia, sympathetic ophthalmia	Corneal edema	Surgical removal
Goel et al. [14]	Female, 60 years old	Vitrectomy, previous posterior chamber IOL removed, scleral fixated IOL,	No corneal edema	Watch & wait approach. Implant dissolved
Srinivasan et al. [15]	Male, 60 years old	Vitrectomy, scleral fixated IOL	Corneal edema, Descemet’s folds	Pharmacologic mydriasis and patient posturing managed to displace it back to the vitreous
Kocak et al. [16]	Female, 72 years old	Long history of uveitis, pseudophakic with intact posterior capsule	No corneal edema or IOP rise	Watch & wait. Implant dissolved
Pacella et al. [17]	Female, 83 years old	Previous ozurdex implant, scleral fixated IOL	Corneal edema	Implant repositioning with BSS injection into the AC
Majumder et al. [8]	Male, 59 years old	Aphakia, vitrectomy	No complications	Observation. Implant dissolution
Kayıkçıoğlu et al. [11]	Patient 1: Male, 63 years old Patient 2: Female, 60 years old Patient 3: Male, 61 years old Patient 4: Female, 79 years old Patient 5: male, 73 years old Patient 6: Male, 70 years old	1: Posterior capsular rupture, zonular dialysis, PPV, sulcus IOL 2: PPV and sutured IOL 3: Diabetic retinopathy, PPV, PC IOL 4: PPV, sutured IOL 5: PPV, anterior vitrectomy, scleral fixated IOL 6: PPV, scleral fixated IOL	1: permanent bullous keratopathy 2: corneal edema and retinal detachment 3: corneal edema 4: no complications 5: bullous keratopathy 6: bullous keratopathy, rise in IOP	1: implant aspiration and keratoplasty 2: pupil dilation, corneal manipulation, patient posturing (reverse Trendelenburg) 3: surgically repositioned using 23 g catheter 4: pupil dilation, corneal manipulation, patient posturing (reverse Trendelenburg) 5: surgical repositioning but then aspirated after re-migration. Keratoplasty 6: aspiration of implant and keratoplasty
Stewart [18]	Male, 43 years old	Posterior uveitis	No complications	Removal via a technique which involved injection of viscoelastic and corneal incision depression
Ha et al. [19]	Patient 1: Male, 64 years old Patient 2: Male, 49 years old	1: Zonular dialysis, PPV, Scleral fixated IOL 2: Defective lens capsule, PPV, Scleral fixated IOL	1: corneal edema 2: corneal edema	1: repositioning with globe massaging 2: repositioning with globe massaging but re-migrated 3 times. Surgical removal was used eventually
Kang et al. [20]	Patient 1: female, 51 years old Patient 2: Male, 50 years old Patient 3: Male, 72 years old Patient 4: Male, 73 years old	1: vitrectomy, sulcus IOL, previous uncomplicated ozurdex 2: vitrectomy, posterior iris claw IOL, previous uncomplicated ozurdex 3: vitrectomy with membrane peel, PC-IOL, YAG capsulotomy, multiple previous ozurdex implants 4: vitrectomy, sulcus IOL, previous uncomplicated ozurdex implant	1: corneal edema, Descemet’s membrane folds 2: corneal edema 3: corneal edema 4: corneal edema	1: surgical repositioning using paracentesis and Sinskey hook 2: surgical repositioning using paracentesis and Sinskey hook 3: surgical removal via an 18-gauge needle and Sinskey hook 4: two surgical re-positionings and then eventually surgical removal due to re-migration
Kishore et al. [21]	Female, 89 years old	Intermediate uveitis	Increase in IOP	Pharmacologic mydriasis and postural relocation achieved re-migration to vitreous
Ku et al. [22]	Female, 75 years old	Three previous uncomplicated ozurdex implants, previous multifocal IOL replaced by Artisan IOL, arcuate keratotomies and corneal suturing to correct astigmatism	Corneal edema	Surgical removal using a 16G IV cannula
Lee et al. [23]	Female, 50 years old	Two previous uncomplicated ozurdex implants	Raised IOP	Surgical removal with Sinskey hook
Nguyen et al. [24]	Female, 58 years old	Retinal detachment, Proliferative vitreoretinopathy	Corneal edema	Surgical removal using 23 *g* needle
Rivera-Perez de Rada [25]	Female, 78 years old	Pseudoexfoliative syndrome, previous IOL and capsule subluxation, scleral fixation IOL	Corneal edema	Postural relocation, ocular massage
Depla et al. [26]	Female, 59 years old	Previous ozurdex with intact posterior capsule at the time, retropupillary iris claw IOL, complicated vitreoretinal surgery	Corneal edema	Surgical removal via 19 *g* needle aspiration and use of viscoelastic
Rahimy et al. [27]	Four patients with mean age 75. two Females and two Males	× 2 BRVO, × 2 chronic noninfectious posterior uveitis × 1 in the bag IOL, × 1 sulcus IOL, × 1 scleral fixated IOL, × 1 AC IOL. × 3 PPV	Not mentioned	surgical removal: corneal incision and reorientation of implant perpendicular to incision. Then viscoelastic injection distal to implant- implant which caused egression
Ruiz-Casas et al. [28]c	Male, 64 years old	Dislocated lens extracted, vitrectomy, anterior chamber IOL	Corneal edema	Surgical removal, using a lens injector to capture and remove Ozurdex
Vela et al. [29]	Female, 65 years old	Multiple retinal detachment surgeries, iris-claw IOL	No corneal edema	Surgical repositioning of implant to VC using 30 g needle. No viscoelastic used
Röck et al. [6]	Patient 1: Female, 47 years old Patient 2: Male, 76 years old Patient 3: Female, 84 years old Patient 4: Female, 69 years old	1: Noninfectious chronic uveitis, vitrectomy, previous dislocated IOL, scleral fixation IOL 2: pseudoexfoliation syndrome, previous dislocated IOL, vitrectomy, scleral fixation IOL, Irvine-Gass syndrome 3: vitrectomy and scleral fixation IOL, Irvine-Gass syndrome 4: noninfectious chronic uveitis, posterior chamber IOL, partial zonular dehiscence so then vitrectomy and surgical posterior capsulotomy done	1: Corneal edema 2: Corneal decompensation with bullous keratopathy 3: Corneal edema, bullous keratopathy 4: Corneal edema, Descemet’s folds	1: Surgical removal via paracentesis with viscoelastic injection and use of 20 g alligator forceps 2: Surgical removal via paracentesis with viscoelastic injection and use of 20 g alligator forceps 3: DMEK 4: surgical removal
Bansal et al. [30]	Patient 1: Male, 47 years old Patient 2: Male, 13 years old Patient 3: Male, 15 years old	1: chronic anterior uveitis, post-lensectomy-vitrectomy, aphakia 2: Bechet’s syndrome, post-lensectomy-vitrectomy, aphakia 3: Chronic anterior uveitis, post-lensectomy and vitrectomy, aphakia	1: Corneal edema, Descemet’s folds 2: Corneal edema, elevation of IOP 3: No complications	1: remigrated to vitreous cavity on its own 2: surgical removal 3: remigrated back to vitreous upon supine position
Khurana et al. [31]	Patient 1: Male, 65 years old Patient 2: Female, 49 years old Patient 3: Male, 61 years old	1: CRVO, PPV, anterior chamber IOL. Ozurdex fragmented 2: noninfectious posterior uveitis, PPV, PCIOL, previous uncomplicated ozurdex implant 3: chronic intermediate uveitis, PPV with membrane peel, PCIOL, zonular dehiscence of lens capsule, YAG capsulotomy, Retisert implantation. The patient has had × 2 times ozurdex implants both resulting in anterior migration	1: Corneal edema 2: Corneal edema, Descemet’s folds 3: After the first implant no corneal edema. After the second implantant corneal edema	1: failed surgical removal resulting into further fragmentation. Fragments eventually migrated to vitreous 2: Surgical removal via viscoelactic and Sinskey hook to reposition the implant and remove using tying forceps 3: After the first implantation, YAG laser broke up implant and remaining part fell back into VC. After the second implantation, the YAG laser used to break up implant into fragments which remained in inferior angle but eventually resolved. Patient had Descemet’s stripping endothelial keratoplasty and a Retisert implant
Chang et al. [32]	Female, 46 years old	Three prior uncomplicated ozurdex implants, bilateral congenital glaucoma, PPV, Baerveldt tube, IOL complicated but its prolapse into the sulcus required repositioning	Corneal edema	Surgical removal via vitrectomy
Eadie et al. [33]	Male, 48 years old	CRVO, previously uncomplicated ozurdex implant, steroid induced glaucoma and trabeculectomy	Superficial punctate keratitis with no edema	Watch & wait. Implant dissolved
D Kumar et al. [34]	Patient 1: Male, 61 years old Patient 2: Male, 57 years old	1: glued IOL 2: glued IOL	1: corneal edema 2: corneal decompensation	1: not specified 2: surgical removal via no-touch technique, the implant aligned perpendicularly with incision and explanted with visco-expression by counter pressure on the posterior lip. PDEK was also required later
A Kumar et al. [35]	Patient 1: Male, 65 years old Patient 2: Male, 54 years old	1: Vogt Koyanagi Harada syndrome, previous PC-IOL complicated by dislocation and replaced by glued Scleral fixated IOL 2: previous complicated cataract surgery due to posterior capsular rupture, then PPV and scleral fixated IOL	1: corneal edema, Descemet’s folds 2: corneal edema	1: Initially mydriasis and postural relocation. Then recurrence was managed with surgical removal 2: Initially mydriasis and postural relocation. A subsequent recurrence was managed with surgical removal
Madi et al. [36]	Male, 53 years old	Idiopathic chronic uveitis, anterior vitrectomy, sulcus IOL which dislocated and thus exchanged for an AC-IOL with peripheral iridotomy. Later developed pseudophakic bullous keratopathy and so had DSAEK and scleral fixated IOL	Corneal graft decompensation	Surgical removal with a Simcoe cannula. Needed repeat DSAEK
Majumbar et al. [37]	Male, 67 years old	Intact PC-IOL	Corneal edema	Surgical removal with the help of viscoelastic substance
Marchese et al. [38]	Male, 77 years old	CRVO, vitrectomy via PPV, sutured scleral-fixated IOL	Increase in IOP, corneal edema	Supine posturing failed to remigrate implant to posterior segment, surgical approach fragmented the implant and irrigation/aspiration probe used to aspirate them
Chen et al. [39]	Male, 58 years old	Diabetic macular edema, vitrectomy via PPV, ACIOL	Corneal edema	Surgically relocated implant in posterior segment using grasping forceps and spatula
Stavrakas et al. [40]	Female, 78 years old	Vitrectomy via PPV, sutureless scleral fixation Carlevale IOL	Corneal edema	Surgical removal

## Discussion

The biodegradable dexamethasone implant Ozurdex^®^ has been designed for use in patients suffering from macular edema secondary to CRVO, BRVO, diabetes, and non-infectious uveitis.

Jirarattanasopa et al. [41] demonstrated the effectiveness of Ozurdex in improving the best corrected visual acuity and the central retinal thickness in patients suffering from macular edema secondary to retinal vein occlusion (RVO) or diabetic retinopathy (DR). The greatest improvement was found in RVO patients and in treatment naïve eyes than in previously treated eyes. Scarammuzi et al. [42] also noted that the use of Ozurdex in diabetic macular edema (DME) can result in long term meaningful benefits while avoiding the significant side effects expected after intraocular corticosteroid injections. Likewise, Massa et al. [43] showed that Ozurdex use in macular edema secondary to non-infectious uveitis provides promising results both in terms of improving visual acuity as well as macular anatomy.

Furthermore, recent studies have emerged, arguing that Ozurdex can also be used for other pathologies such as macular edema arising after vitrectomy or cataract surgery [44, 45] or even exudative AMD [46].

A rare complication of Ozurdex implant is anterior migration from the vitreous cavity to the aqueous chamber. Several large scale studies conducted have shown the overall prevalence of this complication varying from 0.63% to 1.60%, with the incidence being significantly higher in eyes affected by established risk factors [5, 6]. The common risk factors for implant migration include aphakic or pseudophakic eyes, history of vitrectomy, reconstructed iris, zonular dehiscence and open or defective lens capsule [3–5, 7–9, 11].

The vitreous chamber which under normal circumstances would “hold” the implant in place is filled with aqueous humor if an eye is vitrectomized, allowing the implant to move. This state allows the implant to migrate anteriorly unopposed through the pupil especially in aphakic eyes. Even in pseudophakic eyes the implant can circumvent the pupil and reach the aqueous chamber if there is zonular weakness, disrupted posterior lens capsule or pass through an iridotomy if one is present [6, 20].

The most common and serious complication due to this migration is endothelial decompensation and corneal edema, followed by rise in IOP. The underlying mechanism of corneal edema and endothelial decompensation formation is proposed to be associated either to the chemical toxicity of the components of the implant (Dexamethasone, glycolic acid, lactic acid) or due to direct mechanical trauma to the cornea. The risk of occurrence of corneal edema increases in cases of earlier migration (< 3 weeks) compared to the ones that occur later and also in cases where the corneal endothelial cell count is already reduced [30, 31].

In terms of management of the anterior migration of Ozurdex, several options exist and depend on the surgeon’s preference, presence of corneal damage and history of previous migrations after repositioning the implant [9, 19, 20].

In the cases reviewed, the option of observing with close monitoring until the implant dissolves was followed solely in circumstances were there was no corneal edema or rise in IOP [3, 8, 14, 16]. A very common management option was that of implant repositioning to the vitreous chamber, achieved either conservatively or surgically. This option however, carries the risk of the implant remigrating back to the AC [9]. The non-operative technique involves application of a mydriatic agent followed by posturing and positioning the patient’s globe in such a way that the implant becomes dislodged from the anterior chamber and with the help of gravity re-migrates back into the vitreous chamber. This technique may be aided with corneal manipulation using a cotton tip applicator or ocular massage [11, 15, 19, 21, 25]. On the other hand, the operative methods to reposition the implant can vary. In many cases, a limbal incision was made and after insertion of viscoelastic material to protect the anterior chamber structures, the surgeon using a 30-gauge needle injected balanced salt solution to obtain implant repositioning to the vitreous cavity. Other surgeons chose to dislodge the implant from the anterior chamber using a paracentesis then using a Sinskey hook position it near the pupil margin and then through the ruptured posterior capsule back behind the IOL. A common step amongst various techniques was the use of miotic agents such as Miochol after the repositioning to help prevent recurrence of anterior migration [11, 17, 20, 29].

Finally, the course of action selected can be that of surgical removal of the implant, which is particularly common, in cases of severe corneal edema, rise in IOP or multiple failed repositioning attempts. Various techniques for removal have been described. The use of a needle attached to a syringe to aspirate the implant is the most commonly used. During this technique a paracentesis is made and a needle (usually 23-Gauge) is advanced into the anterior chamber. The dexamethasone implant is then aligned with the axis of the needle and as suction is applied, the implant is effectively grasped and eventually pulled out of the eye [1, 11, 24, 26]. Other surgical techniques include use of vitrectomy instruments and forceps [47], use of a lens injector [28] or an IV cannula [22] all of which aim to capture the implant and advance it out of the anterior chamber paracentesis. However some other approaches prioritize minimization of contact between instruments and the implant in order to reduce the chance of breaking it into fragments [12]. These include use of BSS to direct flow through a corneal incision and out of the AC [4, 10] and similarly a no-touch technique using viscoelastic to allow implant egression. In this no-touch technique, a corneal incision is made and the implant is positioned parallel to the incision’s axis. A viscoelastic cannula is advanced distal to the implant and while viscoelastic is injected into the AC, the corneal incision is depressed to direct the flow of viscoelastic out of the incision and thus encouraging the implant to flow outwards with it. This has been proposed as a low risk, cost effective technique [18, 27].

## Conclusion

Migration of Ozurdex in the anterior chamber although rare is a complication that can cause severe damage to the affected eye mostly due to corneal edema and decompensation with eventual need for corneal transplant. Prompt detection and management of such complication is needed. A variety of management options exist which can either involve an operation or just use of maneuvers to reposition the implant back in the vitreous cavity.

## Data Availability

The data used or analysed during the current study is available from the corresponding author on reasonable request.

## Abbreviations

CRVO: Central retinal vein occlusion
BRVO: Branch retinal vein occlusion
IOL: Intraocular pressure
AC: Anterior chamber
VC: Vitreous chamber
PPV: Pars plana vitrectomy
IOL: Intraocular lens
PCIOL: Posterior chamber intraocular lens
SFIOL: Scleral fixated intraocular lens
DMEK: Descemet membrane endothelial keratoplasty
DSAEK: Descemet stripping automated endothelial keratoplasty
SFPCIOL: Sutureless scleral-fixated posterior chamber intraocular lens
ACIOL: Anterior chamber intraocular lens
PI: Peripheral iridotomy
